# HIPK3 Inhibition by Exosomal hsa-miR-101-3p Is Related to Metabolic Reprogramming in Colorectal Cancer

**DOI:** 10.3389/fonc.2021.758336

**Published:** 2022-01-13

**Authors:** Lihuiping Tao, Changliang Xu, Weixing Shen, Jiani Tan, Liu Li, Minmin Fan, Dongdong Sun, Yueyang Lai, Haibo Cheng

**Affiliations:** ^1^ Jiangsu Collaborative Innovation Center of Traditional Chinese Medicine Prevention and Treatment of Tumor, Nanjing University of Chinese Medicine, Nanjing, China; ^2^ The First School of Clinical Medicine, Nanjing University of Chinese Medicine, Nanjing, China; ^3^ School of Integrated Chinese and Western Medicine, Nanjing University of Chinese Medicine, Nanjing, China; ^4^ School of Medicine & Holistic Integrative Medicine, Nanjing University of Chinese Medicine, Nanjing, China

**Keywords:** colorectal cancer (CRC), exosomes, metabolic reprogramming, hsa-miR-101-3p, homeodomain-interacting protein kinase (HIPK3)

## Abstract

**Background:**

Exosomes are extracellular vesicles secreted by most cells to deliver functional cargoes to recipient cells. MicroRNAs (miRNAs) constitute a significant part of exosomal contents. The ease of diffusion of exosomes renders them speedy and highly efficient vehicles to deliver functional molecules. Cancer cells secrete more exosomes than normal cells. Reports have showed that exosomal miRNAs of cancer cells facilitate cancer progression. Yet the complexity of cancer dictates that many more functional exosomal miRNAs remain to be discovered.

**Methods:**

In this study, we analyzed miRNA expression profiles of tissue and plasma exosome samples collected from 10 colorectal cancer (CRC) patients and 10 healthy individuals. We focused on hsa-miR-101-3p (101-3p), a profoundly up-regulated miRNA enriched in plasma exosomes of patients bearing CRC. We performed target analysis of 101-3p and pursued functional studies of this microRNA in two colorectal cancer cell lines, namely HCT116 and SW480.

**Results:**

Our results indicated that inhibiting 101-3p slowed cell growth and retarded cell migration *in vivo* in two colorectal cancer cell lines. Target analysis showed that Homeodomain-interacting protein kinase (HIPK3) is a target of miR-101-3p. HCT116 and SW480 cells stably overexpressing HIPK3 showed increased level of phosphorylated FADD, as well as retarded cell growth, migration, and increased sensitivity to 5-FU. In-depth analysis revealed increased mitochondrial membrane potential upon HIPK3 overexpression along with increased production of reactive oxygen species, number of mitochondria, and expression of respiratory complexes. Measurements of glycolytic parameters and enzymes revealed decreased level of glycolysis upon HIPK3 overexpression in these two cell lines. Xenograft model further confirmed a profoundly improved potency of the synergistic treatment combining both 5-FU and 101-3p inhibitor compared to 5-FU alone.

**Conclusion:**

This study unraveled an oncogenic nature of the exosomal 101-3p and suggested a relationship between the 101-3p-HIPK3 axis and metabolic homeostasis in colorectal cancer. Expression level of 101-3p is positively correlated with glycolytic capacity in CRC and therefore 101-3p itself is an oncomiR. Combining 101-3p inhibitor with chemotherapeutic agents is an effective strategy against CRC.

## Introduction

Exosomes are membrane-bound extracellular vesicles harboring biological components such as lipids, proteins, and nucleic acids (1–3). Diffusion of exosomes in biological fluids and merging of exosomes with recipient cells *via* membrane fusion serves as a means to deliver functional biological molecules to target cells. MicroRNAs (miRNAs), which are a class of small non-coding RNAs, have been identified in the exosomes. miRNA expression profile of exosomes differ greatly from their derivative cells. Active sorting of miRNAs into exosomes reflects various purposes. Reports have shown that exosomes can alter gene expression landscape of target cells through miRNA to achieve various purposes, e.g. formation of pre-metastatic niche (4–6).

Cancer cells secrete larger amount of exosomes than normal cells (7, 8). Exosomes derived from cancer cells are able to reshape both local and distant microenvironments, favoring cancer progression and metastasis. One of the first moncomiRs, miR-21, is discovered in abundance in exosomes of many tumors (9–13). SW480-derived exosomes contain large amount of miR-21 which has the potential to inhibit PDCD4, an apoptosis mediator (14). Other studies have reported that several exosomal miRNAs, e.g. miR-21, miR-1246, and miR-23a, are of potential diagnostic value for colorectal cancer (15).

In this study we discovered a highly upregulated miRNA in plasma exosomes of CRC patients, hsa-miR-101-3p (101-3p). 101-3p was upregulated 8-fold in plasma exosomes of CRC patients compared to healthy individuals. Interestingly, analysis of 101-3p expression in tumor and paired paracancerous tissues of the same CRC patients showed no significant difference, which indicates selective packaging of 101-3p into exosomes. Functional study of 101-3p revealed its oncogenic potential. Bioinformatic analysis, qPCR screening and dual luciferase reporter assay revealed homeodomain-interacting protein kinase 3 (HIPK3) is a direct target of 101-3p. Overexpression of HIPK3 in HCT116 and SW480 cells mimics the effects of inhibiting 101-3p. Further experiments showed that stable HIPK3 overexpression sensitized these two cell lines (^HIPK3+^HCT116 and ^HIPK3+^SW480) to 5-FU-induced apoptosis. JC-1 staining revealed an increase in mitochondrial membrane potential (MMP) in these cell lines compared to their NC counterparts, suggestive of enhanced mitochondrial functionality. However, cytochrome c staining at 12 h post 5-FU treatment showed elevated expression and release of the apoptosis marker. Further screening indicated that increase in VDAC1 expression, a pore-forming protein which facilitates cytochrome C release, accounted for the rise in cytochrome c release and therefore augmented sensitivity to 5-FU induced intrinsic apoptosis. Mitochondrial characterization showed an increase in the number of mitochondria as well as heightened expression of respiratory enzymes required for aerobic respiration in these two HIPK3-overexpressing cell lines. An increase in ROS production was also observed in these two engineered cell lines. Analysis on glycolysis showed that glycolytic parameters, e.g. lactate production and expression of key glycolytic enzymes, all dropped due to HIPK3 overexpression. Further reconstitution experiments confirmed that co-culturing of exosomes derived from wild type CRC with HIPK3-overexpressing cell lines partially restored their oncogenic potential. Xenograft experiments suggest that a combinatorial treatment of both 5-FU and 101-3p inhibitor is a more efficacious therapy than 5-FU alone in colorectal cancer. Our findings unraveled a relationship between 101-3p/HIPK3 axis with metabolic reprogramming in colorectal cancer. To elaborate, because 101-3p did not affect circHIPK3 expression, HIPK3 inhibition by 101-3p is positively correlated to glycolytic capacity in colorectal cancer. This relationship might be bridged by phosphorylated FADD (pFADD). Overexpression of HIPK3 traps a metabolically reprogrammed state in colorectal cancer where its metabolism shifts from glycolysis to oxidative phosphorylation. Clinically, this metabolic shift caused by HIPK3 overexpression weakens tumor and sensitizes it to chemotherapy.

## Methods and Materials

### Clinical Samples and miRNA Sequencing

Tumor and paracancerous tissue pairs were acquired from ten CRC patients at the Jiangsu Province Hospital of Chinese Medicine. Tissue samples were immediately frozen in liquid nitrogen at the time of surgery and stored at -80° C. All experiments were performed in accordance with The Code of Ethics of the World Medical Association (Declaration of Helsinki) and approved guidelines of the Nanjing University of Chinese Medicine. The clinical features of the patients are listed in **Additional File 1: Table S1**. Peripheral blood samples (at least 2.4 mL each) were acquired from these ten patients and another ten healthy individuals as controls. All patients were aware of the use of their samples and have provided us with written consent. All procedures regarding sample preparations were approved by the Ethics Committee of Nanjing University of Chinese Medicine. Plasma exosome purification, RNA extraction of tissue and exosomes, and miRNA sequencing were performed by LC Sciences (Hangzhou, Zhejiang, China). Clinical information of ten CRC patients were provided in **Supplementary Table S1**.

### Cell Lines

Colorectal carcinoma cell lines, HCT-116, SW480, LoVo, HT29, SW620, and a normal colon epithelial cell line FHC were purchased from American Type Culture Collection and cultured in DMEM medium (KeyGen Biotechn, Nanjing, China) supplemented with 10% (v/v) fetal bovine serum (FBS, Gibco) and 1% (v/v) penicillin-streptomycin (Gibco).

### Establishment of Stable HCT116 and SW480 Cell Lines Expressing 101-3p Inhibitor

Short hairpin sequence containing NC (TTCTCCGAACGTGTCACGT) and inhibitor sequence (TTCAGTTATCACAGTACTGTA) of 101-3p were cloned into LV2N (U6/Puro) (GenePharma, Shanghai,China) vector. Constructed LV2N, along with other packaging vectors pGag/Pol, pRev, and pVSV-G, were transfected into 293T cells for lentiviral production. Viruses were harvested by ultracentrifugation. HCT116 and SW480 cells were then treated by viruses either expressing NC or 101-3p inhibitor at a MOI of 10. Treated cells were screened by puromycin at a concentration of 1 μg/mL.

### Establishment of Stable HCT116 and SW480 Cell Lines Overexpressing HIPK3

ORF (open reading frame) of HIPK3 were synthesized by Genescript (Nanjing, Jiangsu, China) and cloned into pLV-EF1a-puro. Constructed pLV-EF1a-puro, along with other packaging vectors pGag/Pol, pRev, and pVSV-G, were transfected into 293T cells for lentiviral production. Viruses were harvested by ultracentrifugation. HCT116 and SW480 cells were then treated by viruses either expressing blank (NC) or HIPK3 at a MOI of 10. Treated cells were screened by puromycin at a concentration of 1 μg/mL.

### Target Analysis of 101-3p

To determine possible target genes of 101-3p, four online databases were employed, and each of which produced a list of possible target genes. These databases are Diana tools (microT-CDS), TargetScan 7.2, miRDB, and TarBase V.8. Our screening criteria is as follows: Diana tools (microT-CDS) with a miTG score greater than 0.90; TargetScan 7.2 with at least 2 conserved binding sites; miRDB with a rating greater than 90; Tarbase v.8 with a rating greater than 0.9. The final gene list consists of 15 genes and names of the genes can be found in **Figure 2A**.

### Data Collection and Analysis From the TCGA Database

HIPK3 and 101-3p expression data in CRC patients were acquired from the TCGA database (https://cancergenome.nih.gov). HIPK3 transcript data were acquired from 488 CRC patients and 30 normal cases, while miRNA expression data were acquired from 539 CRC and 9 normal cases. Annotation information was downloaded from genecode (https://www.gencodegenes.org/) and miRBase (http://www.mirbase.org/).

### Cell Proliferation Assay

Proliferation of cells was monitored using the CCK-8 kit (Beyotime, Nantong, China) according to the manufacturer’s instructions. Briefly, 5×10^3^ cells were seeded into each well of 96-well plates. Samples were measured at 48 h post-seeding in triplicate at 450 nm by the Tecan Infinite 200 PRO plate reader (Tecan Group Ltd., Männedorf, Switzerland).

### EdU Incorporation Assay

Cell proliferation was also monitored by EdU (5-ethynyl-20-deoxyruidine) assay using the EdU assay kit (KeyGen Biotech., Nanjing, China). Cells were seeded into each well of 24-well plates, followed by incubation at 37 °C and 5% CO_2_ for 48 h. Cells were then treated with 10 μM EdU for 2 h and then fixated by 4% paraformaldehyde. Cells were then stained with the Click-iT EdU mixture solution. Cell nuclei were then stained with DAPI. Proliferation was quantified as the percentage of cells actively incorporating EdU in a microscopic field according to the manufacturer’s instructions. Images were taken using a Zeiss Axio Vert. A1 microscope.

### Colony Formation Assay

Under the culture condition described before, 1×10^3^ cells were seeded in a six-well plate and cultured for 2 weeks. Then, colonies were washed with phosphate-buffered saline, fixed with methyl alcohol for 10 min, stained with crystal violet (0.5% wt/vol) for 20 min and finally counted under a microscope.

### Wound Healing Assay

Cells were allowed to grow to a 90–95% confluence. A scratch wound was made using a 200 μL pipette tip on the cell layer. Scratches were photographed at 0 and 48 h and widths of scratches were measured digitally. Measurements were taken from assays in triplicates for each type of cells.

### Transwell Assay

About 5x10^4^ cells were allowed to grow in the inset chamber of a 24-well transwell plate (Corning). Cells in the chamber were cultured in 100 μL FBS-free culturing medium with the Matrigel membrane where the chamber itself was submerged into the wells of a plate containing 500 μL complete growth media. After 24 h, cells failed to invade to the lower surface of chamber were wiped by cotton scrubs whereas cells attached to the lower surface were fixated in 4% paraformaldehyde and stained by 0.1% crystal violet. Photographs of invaded cells were taken by a light microscope and number of number of invaded cells were recorded from assays in triplicates for each type of cells.

### Apoptosis Treatment

Different types of cells were either treated by PBS or 5-FU (50 μM, the only concentration used in this study). Treatment periods vary and are indicated in figures.

### Annexin V-Prodium Iodide (PI) Double Staining Using Flow Cytometry

Cells were treated by Annexin V-FITC in Ca^2+^-containing binding buffer at room temperature for 30 min. PI was added 5 min before flow cytometric measurement. Degree of apoptosis was determined by Annexin V (green) and PI (red) fluorescence probed by flow cytometry on a Beckton-Dickinson flow cytometer.

### Exosomes Internalization Assay

PKH67 (Sigma, MINI67–1KT) was used to label exosomes (2 μg) from HCT116 and SW480 isolated by differential centrifugation according to manufacturer’s instructions. To visualize exosome transfer, PKH-67-labeled exosomes were cultured with FHC cells for 4h. 4’, 6-diamidino-2-phenylindole (DAPI) (abcam, ab104139) was used for nuclear staining, while the cytoskeleton of MGC803cells was selectively stained with TRITC Phalloidin (KeyGEN, KGMP0012). Internalization of exosomes was probed by a confocal laser scanning microscope (Leica TCS SP8, Leica Microsystems)

### Dual-Luciferase Reporter Assay

Fragments (445 bp) of 3’-UTR of HIPK3 containing either the 101-3p binding site (position 1677-1683 of the 3’-UTR of HIPK3) or mutated 101-3p binding site were cloned into pSI-check2 plasmid (Hanbio, Shanghai, China). Plasmids were the transfected into 293T cells. 101-3p mimic or NC were added to transfected 293T cells. hRLuc (Renilla luciferase) and fLuc (Firefly luciferase) were detected by Promega Dual-Luciferase system kit (Promega, Beijing, China) on a Tecan Infinite 200 PRO plate reader.

### Mitochondrial Membrane Potential Determination

The JC-1 mitochondrial membrane potential kit (KeyGen Biotech., Nanjing, China) was employed to measure mitochondrial membrane potential in both wild type and HIPK3 over-expressing cells subjected to different treatment. Briefly, 1 mL of 10 µg/mL JC-1 was added to DMEM medium in 6-well plates and the mixture was then incubated for 20 min at 37°C in an incubator supplemented with 5% CO_2._ Red (Ex=514 nm, Em=529 nm) and green fluorescence (Ex=585 nm, Em=590 nm) of treated Jurkat cells were measured on a flow cytometer (FACS Calibur; BD Biosciences). Proportion of cells in the Red-/Green+ quadrant was calculated. Other cells were grown on coverslips and fixated by 4% paraformaldehyde for 2 h to be observed by a Zeiss Axio Vert.A1microscope equipped with an Olympus X-cite 120 Q light source.

### Determination of ROS Generation

The ROS detection kit (KeyGen Biotech., Nanjing, China) was used to measure ROS production of cells. Briefly, 2’,7’ - dichlorofluorescein diacetate (DCFH-DA) was diluted to a concentration of 10 μM using DMEM medium. 1x10^6^ cells were collected and resuspended in medium containing DCFH-DA, followed by incubation at 37°C for 20 min. Green fluorescence of cells was detected by flow cytometry. Other cells were grown on coverslips and fixated by 4% paraformaldehyde before DCFH-DA treatment. Fluorescence images were taken by a Zeiss Axio Vert.A1microscope equipped with an Olympus X-cite 120 Q light source.

### Western Blot Analysis

Cells were collected and washed twice with ice-cold PBS, followed by lysis using RIPA buffer (protein inhibitor included) for 40 min. Lysed cells were precipitated by centrifugation (12,000 rpm for 10 min at 4°C) and cell extracts in the supernatant were harvested. Proteins in the supernatant were separated using 12% SDS-PAGE and transferred to PVDF membranes (Millipore, Bedford, MA, USA). Membranes were then blocked in non-fat 5% milk at room temperature for 1 h and blotted with appropriate primary antibodies at a dilution ratio of 1:1000 at 4°C overnight. Following three washes in PBS (10 mM, pH 7.4) containing 0.1% Tween 20, membranes were blotted with secondary antibodies of either mouse or rabbit origin at a dilution ratio of 1:2000 for 1 h at room temperature. After three washes in PBS with 0.1% Tween 20, the blots were visualized using ECL detection reagents. Bands were quantified by the ImageJ software.

### Exosomes Isolation From Culture Medium

Cells were cultured in the DMEM medium supplemented with 10% exosome-free FBS. Cell culture supernatant was centrifuged after 2 days of culturing and centrifuged at 300 g for 10 min twice before ultracentrifugation at 100,000 × g for 120 min to collect the exosomes.

### RNA Isolation and Quantitative Real-Time

Total RNA of cells was extracted using the Trizol reagent (Invitrogen, Carlsbad, USA) according to the manufacturer’s instructions. Reverse transcription was implemented using either a routine reverse transcription kit or a miRNA 1st Strand cDNA Synthesis Kit (Vazyme, Nanjing, China). Quantitative real-time PCR was performed using the AceQ qPCR SYBR Green Master Mix (Vazyme, Nanjing, China). The PCR cycling conditions were as follows: an initial start at 95°C for 10 min, followed by 40 cycles at 95°C for 15 s and 60°C for 1min. mRNA levels of various genes were expressed as a ratio to the internal control GAPDH or U6.

### Cytochrome C Staining

Cells were washed three times with PBS buffer (pH7.4). For flow cytometry, cells were digested by 0.25% trypsin and resuspended. Cells were then fixated by 4% paraformaldehyde on ice for 2 h, followed by treatment by 0.1% Triton solution for membrane piercing for 30 min on ice with occasional shaking. Cells were again washed by PBS three times before blocking by 1% BSA solution for 30 min on ice. Cells were then washed by PBS and incubated with cytochome C-FITC antibody on ice in dark. DAPI was added 5 min before flow cytometric measurement or microscopic observation. Resuspended cells were probed by flow cytometry on a Beckton-Dickinson spectrometer while others were observed by fluorescence microscopy on Zeiss Axio Vert.A1microscope.

### Mitochondrial DNA Quantification

Mitochondrial DNA (mtDNA) was quantified as previously described (ref.). Briefly, total genomic DNA of cells was extracted using the Genomic DNA mini preparation Kit (Beyotime, Beijing, China). Mitochondrial DNA level was determined based on the relative level of cytochrome oxidase 1 compared to β-globin (Primers in **Supplementary Material**).

### Co-Culturing of Wild Type Exosomes With Stable HCT116 and SW480 Cell Lines Overexpressing HIPK3

For rescue experiments, ^HIPK3+^HCT116 and ^HIPK3+^SW480 cells were co-cultured with their respective wild type exosomes. Briefly, 2 x 10^6^ cells mutant cells were co-cultured with excessive exosomes derived from 4 x10^6^ wild type cells, at which exosomes were collected by ultracentrifugation.

### Determination of Glucose Consumption, and ATP, Acetate, Pyruvate and Acetyl-CoA Production

Cells of different treatment groups were cultured in FBS-free medium overnight prior to measurement. Glucose consumption, lactate, ATP, pyruvate and Acetyl-CoA production assay kits were all purchased from Solarbio (Beijing, China) and used according to manufacturer’s instructions. All experiments were performed in triplicates.

### Immunohistochemistry (IHC)

Formalin-fixed and paraffin-embedded tissues were processed into thin sections and deparaffinized before antigen retrieval using 10 mM citrate buffer (92°C for 30 min). Gene expression was evaluated according to stain intensity and the percentage of positive cells. The intensity of staining was graded as 1 = weak staining, 2 = moderate staining and 3 = strong staining. The percentage of stained cells was graded as 0 = 0-5%, 1 = 6-25%, 2 = 26–50%, 3 = 51-75% and 4 = 75-100%. The final score was obtained by multiplying the two scores.

### Oxygen Consumption Rate

Mitochondrial oxygen consumption rates (OCRs) and extracellular acidification rates (ECAR) were measured using a Seahorse XF24 Extracellular Flux Analyzer (Seahorse Bioscience). Cells were seed at a density of 20,000 cells in XF24 cell culture microplates and were continuously incubated for 12 h in hypoxia. The culture medium was changed to XF base media as recommended by Seahorse Bioscience before measurement. The ATP synthase inhibitor Oligomycin A (2 μM), the ATP synthesis uncoupler carbonyl cyanide-4-trifluoromethoxyphenylhydrazone FCCP (1.5 μM), the complex I inhibitor rotenone (2 μM), and complex III inhibitor antimycin A (2 μM) were used to determine OCR parameters. OCR is shown in pmol/min/10^5^ cells and ECAR is shown in mpH/min/10^5^ cells. All experiments were performed in triplicate.

### Xenograft Experiments

Tumor cells were first grown *in vitro* to multiply and then concentrated to an injection volume of 100 mL PBS containing about 2x10^6^ cells. Injection by syringe was made to the mid-right flank of nude mice to allow xenograft tumor formation. For IHC analysis, injected NC HCT116 and ^HIPK3+^HCT116 cells were allowed to grow *in vivo* without treatments for 30 days. Tumors volume were measured and recorded at regular time intervals, and tumors were excised at day 30 to be photographed.

For intervention experiments, namely 5-FU and lentiviral treatment, wild-type HCT116 cells were used for injection into nude mice and divided into three groups: a control group treated by NC viruses and two experimental groups which received treatment either by 5-FU and virus harboring scramble control, or 5-FU and virus harboring 101-3p inhibitor sequence. Each group contained 6 mice. For the control group, PBS was administered at day 10, 17, 24 intraperitoneally. For treatments that require 5-FU, 5-FU was administered intraperitoneally at day 10, 17 and 24 at a dosage of 20 mg/kg. For treatments that require virus, lentiviruses containing either scramble control or 101-3p inhibitor were administered intratumorally at day 10, 17 and 24 (50 μL containing about 5x10^7^ virus). Tumor volumes were measured and recorded at regular time intervals, and tumors were excised at day 30 to be photographed.

### Statistical Analysis

Data in this study are presented as mean ± SD. Student’s t-test was used for statistical analysis. All statistical analyses were conducted using Graphpad Prism 6. P-value less than 0.05, 0.01, 0.001, and 0.0001 is indicated by *, **, ***, and ****, respectively.

## Results

### hsa-miR-101-3p Is Upregulated in Plasma Exosomes in CRC Patients and Promotes Cell Growth and Migration in CRC Cell Lines

**Figure 1:**
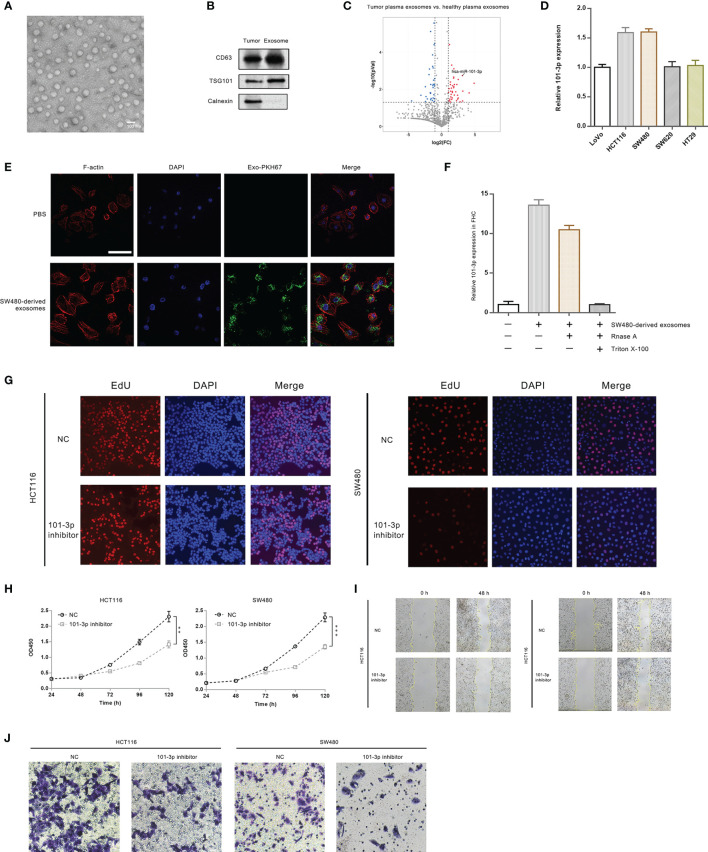
101-3p is highly up-regulated in CRC plasma exosomes and promotes oncogenesis. **(A)** Transmission electron micrograph of exosomes isolated from CRC patients. **(B)** Immunoblots of key proteins of isolated exosomes. **(C)** Volcano plots of miRNA sequencing results: plasma exosomes from CRC patients *vs*. plasma exosomes from healthy individuals. **(D)** Innate expression level of 101-3p in five CRC cell lines. **(E)** Uptake of SW480-derived exosomes by FHC cell monitored by fluorescence microscopy. **(F)** 101-3p level of FHC cells following uptake of SW480-derived exosomes. **(G)** Edu incorporation of HCT116 and SW480 cells stably expressing NC sequence or 101-3p inhibitor sequence. **(H)** Growth curves of HCT116 and SW480 cells stably expressing NC sequence or 101-3p inhibitor sequence monitored by CCK-8. **(I)** Wound healing assay of HCT116 and SW480 cells stably expressing NC sequence or 101-3p inhibitor sequence. **(J)** Transwell assay of HCT116 and SW480 cells stably expressing NC sequence or 101-3p inhibitor sequence. **, *** correspond to a p-value less than 0.01 and 0.001, respectively.

We collected tissue and blood samples from 10 CRC patients and 10 healthy individuals. For tissue samples, tumor and paired paracancerous tissues were collected from CRC patients. Likewise, plasma exosomes in peripheral blood from CRC patients and healthy individuals were collected. A total of 40 samples were analyzed by RNA-Seq (10 paired samples of tumor and paracancerous tissues, 10 samples of plasma exosomes of patients and 10 samples of plasma exosomes from healthy individuals). In this work, we are only publishing miRNA analysis. **Figure 1A** shows a transmission electron micrograph of plasma exosomes collected from one of the CRC patients. **Figure 1B** shows immunoblots of key proteins (i.e. CD63, TSG101 and Calnexin) of tumor and plasma exosomes collected from the same CRC patient.

miRNA data and statistics were compiled and analyzed. Tumor samples and plasma exosome samples of CRC patients exhibited vastly different expression profiles of miRNA. **Figure 1C** shows a volcano plot comparing miRNA expressions in CRC plasma exosomes and healthy plasma exosomes. We pinpointed a miRNA, namely hsa-miR-101-3p (indicated by an arrow in the graph), which was up-regulated about 8-fold in exosomes circulating in plasma of patients bearing colorectal tumors. Relevant analysis of bioinformatics is shown in **Supplementary Figure 1**. Original data can be found in supplementary excel files.

We then examined 101-3p expression in several CRC cell lines, i.e. SW480, HCT116, LoVo, SW620, and HT29 (**Figure 1D**). Our results showed a relatively high expression of 101-3p in HCT116 and SW480.

To show CRC cell-derived exosomes can transfer 101-3p, we co-cultured SW480-derived exosomes with FHC cells. We imaged intake of SW480-derived exosomes by FHC, a normal colon epithelial cell line by confocal microscopy (**Figure 1E**). SW480-derived exosomes were labeled by a green dye, PKH67. It is evident that FHC cells, where were shown by F-actin staining, engulfed the green exosomes. We also probed 101-3p expression in FHC co-cultured with SW480-derived exosomes (**Figure 1F**). Innate expression of 101-3p in FHC cells was very low. After co-culturing with SW480-derived exosomes, a sharp increase in 101-3p expression in FHC was observed. A similar increase of 101-3p was observed in FHC if co-cultured with SW480-derived exosomes pre-treated with RNase A alone, but not with exosomes pre-treated with RNase A and Triton X-100.

To unravel the function of 101-3p, we then treated HCT116 and SW480 cells with lentiviruses expressing 101-3p inhibitor to produce stably transfected cell lines. Stereotypic characterization of these cell lines followed. **Figure 1G** shows that, compared to cell lines treated with NC, inhibition of 101-3p in two engineered cell lines showed retarded cell growth as judged by decreased rate of EdU incorporation in two engineered cell lines. Similar inhibition was observed in CCK-8 experiments shown in **Figure 1H**. Wound healing assays were also performed. Inhibition of 101-3p decreased migration potential of both HCT116 and SW480 cells *in vivo* (**Figure 1I**). Invasion assay using the transwell apparatus showed similar inhibition of invasion potential of both cell lines upon 101-3p inhibition (**Figure 1J**). Our results indicate that inhibition of 101-3p is closely related to decreased of oncogenicity in CRC cell lines. Quantitative analysis of Figure G, I and J can be found in **Supplementary Figures 3A–C**, respectively.

### 101-3p Targets HIPK3 and Inhibits Its Expression

To pinpoint the target gene of 101-3p, we employed four miRNA databases and related softwares, i.e. Diana (microT-CDS), TargetScan release 7.2, miRDB, and TarBase v.8 to narrow down the range of possible target genes of 101-3p. The screening criteria is depicted in Bioinformatic Analysis in the Methods and Materials section. The final list consists of 15 genes.

**Figure 2:**
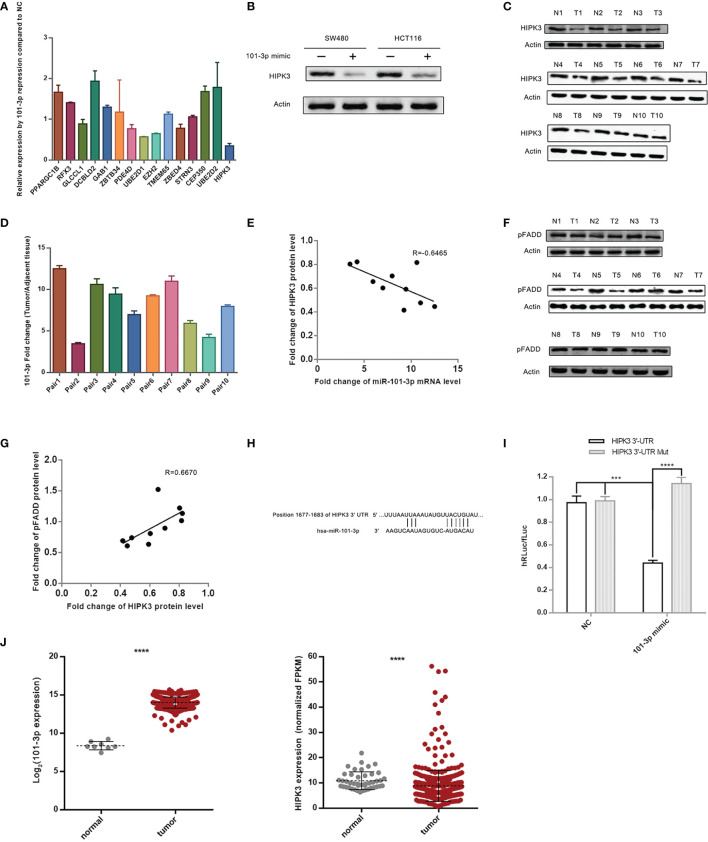
HIPK3 is the target gene of 101-3p. **(A)** Relative expression level of 15 final screened genes in HCT116 following transfection by 101-3p mimic. **(B)** Reduction of HIPK3 at the protein level by 101-3p in both HCT116 and SW480. **(C)** Protein level of HIPK3 in clinical tissue samples. **(D)** Fold change of 101-3p in clinical tissue samples (10 pairs, tumor *vs*. paracancerous tissue). **(E)** Pearson correlation plot of HIPK3 and 101-3p. **(F)** Protein level of phosphorylated FADD (pFADD) in clinical tissue samples. **(G)** Pearson correlation plot of HIPK3 and pFADD. **(H)** Textual representation of the fragment of HIPK3 3’-UTR containing 101-3p binding site. **(I)** Dual-reporter luciferase assay using wild type or mutated 101-3p binding site sequence. Please refer to the methods and materials section for details. **(J)** 101-3p and HIPK3 expressions of deposited data of clinical samples from CRC patients in the TCGA database. **** correspond to a p-value less than 0.0001. *** correspond to p-value less than 0.001.

To precisely determine the target gene of 101-3p, we performed a qPCR screening of these 15 genes following transfection of 101-3p mimic in HCT116 cells. Although miRNAs may exert inhibitory effects *via* translational repression, inhibition through mRNA degradation remains a major mechanism and is worth examining. **Figure 2A** shows that the HIPK3 gene experienced greatest inhibition at the mRNA level among all genes tested. To confirm this result, we transfected 101-3p mimic into HCT116 and SW480 cells and monitored HIPK3 protein level. As shown in **Figure 2B**, 101-3p substantially decreased HIPK3 protein level both in HCT116 and SW480. However, 101-3p mimic did not affect circHIPK3 level in these two cell lines (**Supplementary Figure 3O**).

We monitored HIPK3 protein level and 101-3p level in 10 paired CRC tissue sample (same samples used for miRNA sequencing) leftovers. **Figure 2C** shows immunoblots of HIPK3 in 10 paired CRC tissue samples. **Figure 2D** shows relative 101-3p levels in these samples. A Pearson’s correlation scatter plot is shown in **Figure 2E**. We observed an R value of -0.6465, which indicates a strong inverse correlation connecting 101-3p and HIPK3 in our clinical tissue samples.

A previous study reported that HIPK3 phosphorylates FADD at Serine194 (pFADD) (16). We then probed HIPK3 and pFADD protein level in 10 paired CRC tissue samples. **Figure 2F** shows immunoblots of pFADD. **Figure 2G** shows a Pearson’s correlation scatter plot connecting fold change values of these two proteins. An R value of 0.6670 indicates strong positive correlation between HIPK3 and pFADD.

To further confirm HIPK3 is a target gene of 101-3p, we performed a Dual-Luciferase reporter assay. Fragments of 3’-UTR of HIPK3 containing either the binding site or mutated binding site of 101-3p were cloned into appropriate plasmids and transfected in 293T cells. 101-3p mimics were added to transfected 293T cells. **Figure 2H** shows a textual representation of a fragment of 3’-UTR of HIPK3 containing the binding site of 101-3p. **Figure 2I** shows the degree of reduction based on hRLuc/fLuc ratio. It is evident that 101-3p mimic, not a NC sequence, triggered a substantial reduction of hRLuc in plasmid containing wild type 3’-UTR of HIPK3, but not mutated 3’-UTR of HIPK3. Therefore, HIPK3 is a target gene of HIPK3.

**Figure 2J** shows HIPK3 and 101-3p expressions in tumor and paracancerous samples in the TCGA database. Statistical analysis revealed that HIPK3 expression is lower in tumor samples with a p value less than 0.001, while that of 101-3p is higher in tumor samples with a p value once again less than 0.001.

### Overexpression of HIPK3 Inhibits Tumor Growth and Migration

We then examined the tumor suppressive potential of HIPK3 in two colorectal cancer cell lines, HCT116 and SW480. Two stable cell lines overexpressing HIPK3, namely ^HIPK3+^HCT116 and ^HIPK3+^SW480, were constructed.

**Figure 3:**
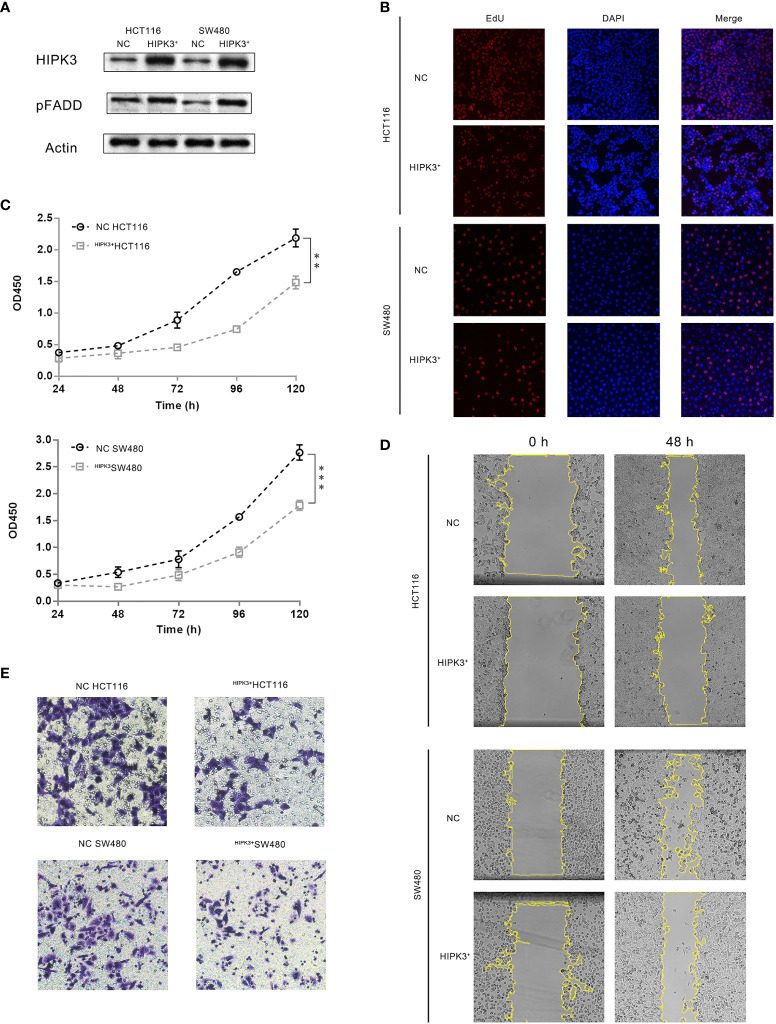
CRC cells stably over-expressing HIPK3 exhibited decreased oncogenic potential. **(A)** Immunoblots of HIPK3, pFADD in HCT116 and SW480 cells stably over-expressing HIPK3. **(B)** EdU incorporation assay of HCT116 and SW480 cells stably over-expressing HIPK3. **(C)** Growth curves of HCT116 and SW480 cells stably over-expressing HIPK3. **(D)** Wound healing assay of HCT116 and SW480 cells stably over-expressing HIPK3. **(E)** Transwell assay of HCT116 and SW480 cells stably over-expressing HIPK3. **, *** correspond to a p-value less than 0.01 and 0.001, respectively.

HIPK3 overexpression and pFADD up-regulation was confirmed by western blot (**Figure 3A**). circHIPK3 was monitored in these two engineered cell lines and no discernable increase or decrease was observed. circRNA-forming Alu repeats are in introns and therefore not found in ORF of HIPK3. EdU incorporation assays showed inhibited growth of the two HIPK3-overexpressing cell lines compared to their NC (cells treated by blank viruses) controls (**Figure 3B**). Inhibited proliferation was also confirmed by growth curves generated from CCK8 assay (**Figure 3C**). Wound healing assay showed dampened migration ability (**Figure 3D**), while transwell assay showed dampened invasion ability of two engineered lines (**Figure 3E**). Quantitative analysis of **Figures 3B, D, E** can be found in **Supplementary Figures 3D–F**, respectively. Results in this section suggest overexpression of HIPK3 decreases oncogenicity of both HCT116 and SW480, and therefore implicate HIPK3’s positive contribution towards tumor suppression.

### Overexpression of HIPK3 Sensitizes Colorectal Cancer Cells to 5-FU

**Figure 4:**
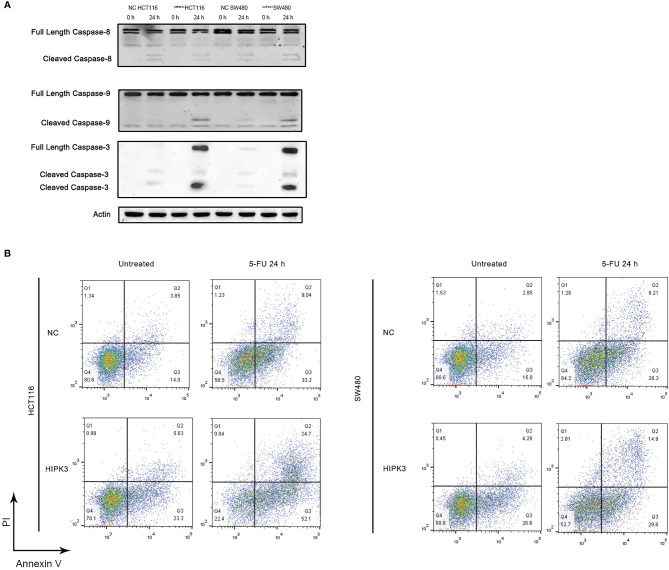
HCT116 and SW480 cells stably over-expressing HIPK3 are more susceptible to 5-FU. **(A)** Caspase 8, 9, and 3 expression of cells treated by 5-FU. **(B)** Flow cytometric analysis of apoptosis using PI/Annexin V dual staining.

Both NC and HIPK3-overexpressing cell lines were subjected to apoptosis treatment by 5-FU, a widely used chemotherapy agent which induces intrinsic apoptosis. Two engineered cell lines were more sensitive to 5-FU than their NC counterparts as both cleaved caspase 9 and caspase 3 expression were considerably higher in HIPK3-overexpressing cell lines (**Figure 4A**, lane 4 and 8). Western blot results were further quantitatively analyzed by the Image J software and graphical representations of caspase levels were shown in **Supplementary Figure 3G**.

Degree of apoptosis was probed by Annexin V/PI double staining and flow cytometry (**Figure 4B**). The first and third columns in **Figure 4B**, the untreated groups (not treated by any apoptosis inducers), show increased degree of apoptosis for the two HIPK3-overexpressing cell lines compared to their NC counterparts. Upon apoptotic treatment by 5-FU for 24h (second and fourth columns), two HIPK3-overexpressing cell lines showed even higher percentage of apoptosis compared to two NC cell lines. Quantitative analysis of **Figure 4B** is shown in **Supplementary Figure 3H**. Flow cytometry results agreed with immunoblotting results, thereby further consolidating the finding that overexpression of HIPK3 enhances intrinsic apoptosis in colorectal cancer cells.

### HIPK3 Overexpression Increases Mitochondrial Membrane Potential

Intrinsic apoptosis is a mitochondrion-mediated process and release of cytochrome C of the mitochondria is a symbolic event in this process (17). Once cytochrome C is released, cells are committed to apoptosis beyond remedy (18). Mitochondrial membrane potential (MMP) is another important parameter positively related to mitochondrial membrane integrity and functionality (19). During the process of apoptosis, MMP drops as the mitochondrial permeability transition pores (mPTPs) open to release cytochrome C. Opening of mPTPs renders mitochondrial membrane depolarized, thus the decrease in MMP. We employed JC-1 staining to probe MMP in our cell lines to seek possible reasons for increased vulnerability to apoptosis in two HIPK3-overexpressing cell lines.

**Figure 5:**
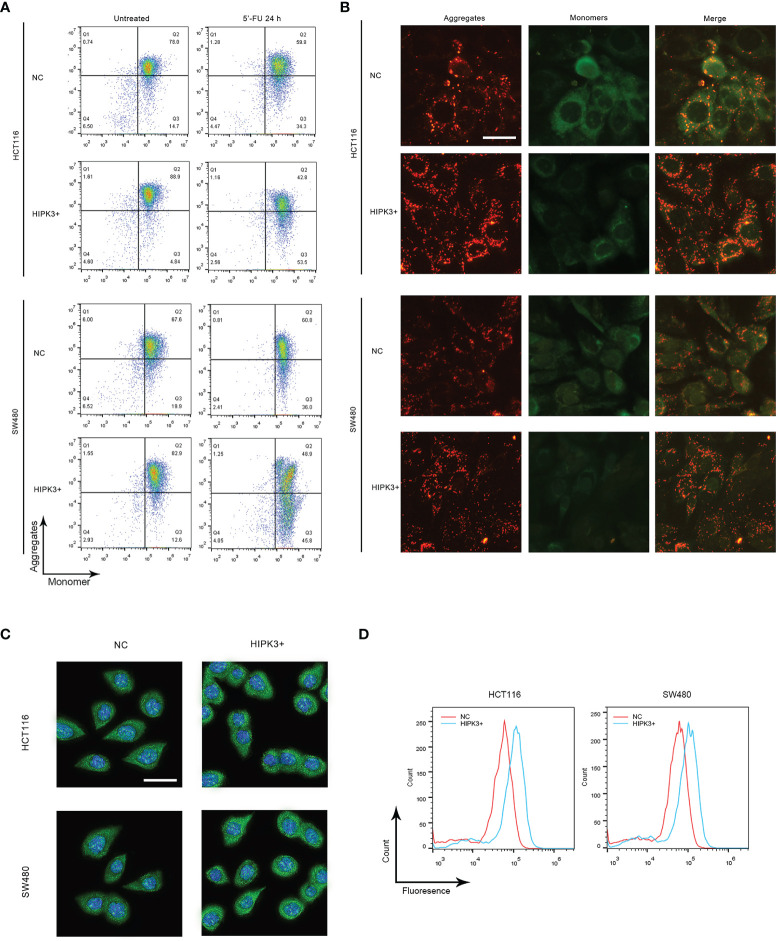
HCT116 and SW480 cells stably over-expressing HIPK3 exhibited alterations in mitochondrial characteristics. **(A)** Mitochondrial membrane potential monitored by flow cytometry using the JC-1 dye. **(B)** Mitochondrial membrane potential probed by fluorescence microscopy. **(C)** Immunofluorescence of cytochrome **(D)** Histogram of cytochrome C generated by flow cytometry.

Flow cytometry (**Figure 5A** left column) results indicate that, if untreated, two HIPK3-overexpressing cell lines exhibited higher MMP compared to their respective controls (more population in the 4^th^ quadrant), which was in contrast to our expectations. However, two HIPK3-overexpressing cell lines displayed lower MMP upon apoptotic treatment by 5-FU compared to their NC controls (**Figure 5A** right column). Fluorescence microscopic images showing JC-1 staining of cells (NOT treated by 5-FU) confirmed flow cytometry results (**Figure 5B**). Two HIPK3^+^-overexpressing cell lines showed more intense staining of the JC-1 dye under natural conditions. To further elaborate, under natural conditions, HIPK3^+^-overexpressing cells exhibited higher MMP compared to control cells, whereas apoptosis triggered a sharper drop in MMP for HIPK3^+^-overexpressing cells and therefore after 24 h of 5-FU treatment, HIPK3^+^-overexpressing cells displayed lower MMP compared to controls. We believe the sharp drop in MMP of the two engineered cell lines can be ascribed to their increased vulnerability to intrinsic apoptosis.

Cytochrome C expression and release is another factor pertinent to mitochondrial membrane integrity. Cells treated by 5-FU for 24 h were stained with fluorescent cytochrome C antibody and probed by both fluorescence microscopy and flow cytometry. As expected, both knockout cell lines showed stronger green fluorescence compared to their respective controls, evident both in the fluorescence images (**Figure 5C**) and flow cytometry histograms (**Figure 5D**). Quantitative analysis of **Figures 5A, C** is shown in **Supplementary Figures 3I, J**, respectively.

### HIPK3 Overexpression Increases VDAC1 Expression

**Figure 6:**
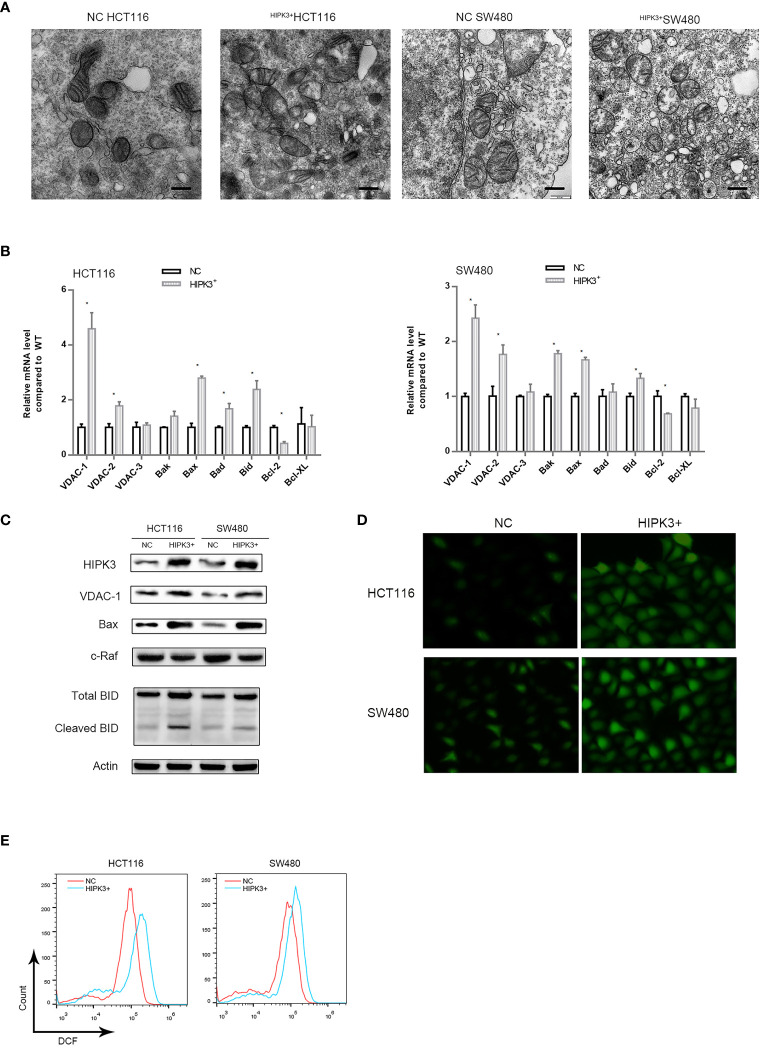
Expression of key proteins related to apoptosis in mitochondria were altered in HCT116 and SW480 cells stably over-expressing HIPK3. **(A)** Transmission electron micrograph of mitochondria. **(B)** mRNA level of key proteins related mitochondria-mediated apoptosis. **(C)** Immunoblots of key enzymes in mitochondrial respiration. **(D)** ROS generation probed by DCFH-DA. **(E)** ROS quantification by flow cytometry (histogram). * correspond to a p-value less than 0.05.

We observed mitochondrial morphology in two engineered cell lines using transmission electron microscopy. **Figure 6A** shows no obvious change in morphological features of mitochondria in two mutant cell lines. However, the number of mitochondria appeared to increase upon HIPK3 overexpression. This may explain the increased MMP in two mutant cell lines.

We further performed a qPCR screen for proteins which may account for this mitochondrial alteration and vulnerability to intrinsic apoptosis (**Figure 6B**). Mitochondrial pore-forming proteins which are components of mPTPs, namely VDAC1,2,3 were included (20–22), so were two other pore-forming and pro-apoptotic proteins, Bak and Bax. Two pro-apoptotic proteins Bad and Bid, as well as two anti-apoptotic proteins Bcl2 and Bcl-XL were also included. qPCR results showed that number of VDAC1, Bax and Bad transcripts was significantly increased in two engineered cell lines while that of Bcl2 was decreased. Protein level detection for VDAC1, BAX and Bad confirmed qPCR results (**Figure 6C**). We also probed the level of a negative regulator of VDAC1, namely c-Raf, which is also a potential oncogene (23). As expected, c-Raf’s expression was decreased in HIPK3-overexpressing cells compared to NC cells. Quantitative analysis of **Figures 6C** is shown in **Supplementary Figure 3K**.

Opening of VDAC1 pores has been linked to heightened mitochondrial Reactive Oxygen Species (ROS) production previously (24). Opening of voltage dependent anion channels promotes reactive oxygen species generation (ROS), mitochondrial dysfunction and cell death in cancer cells. Intake of ADP and Pi through mPTPs and subsequent oxidation *via* mitochondrial oxidative phosphorylation generates ROS. ROS production was probed in all four cell lines. Two HIPK3-overexpressing cell lines exhibited higher ROS production compared to their wild type counterparts, which is evident in both fluorescence microscopic images and flow cytometry measurements (**Figures 6D, E**). Quantitative analysis of **Figure 6E** is shown in **Supplementary Figure 3L**.

### HIPK3 Overexpression Enhances Aerobic Respiration and Weakens Glycolysis in Colorectal Cancer Cells

**Figure 7:**
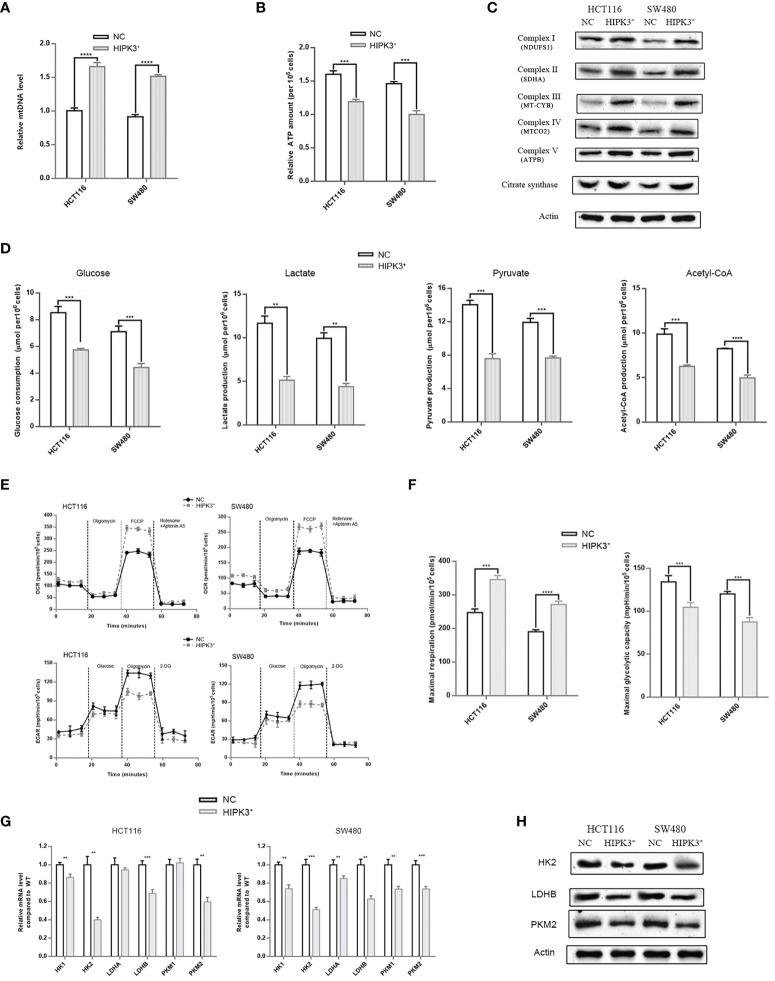
HIPK3 over-expression reduced expressions of glycolytic enzymes while increased expression of mitochondrial enzymes in HCT116 and SW480 cells stably over-expressing HIPK3. **(A)** mtRNA quantification. **(B)** ATP production assay. **(C)** Immunoblots of key enzymes in the respiratory chain. **(D)** Glucose consumption, lactate, pyruvate, and ATP production assay. **(E)** Seahorse OCR and ECAR measurements. **(F)** Quantitative analysis of **Figure 7F** showing maximal respiration and glycolytic capacity. **(G)** mRNA level of key enzymes in glycolysis. **(H)** Immunoblots of key enzymes in glycolysis. **, ***, **** correspond to a p-value less than 0.01, 0.001, and 0.0001, respectively.

We then examined other mitochondrial parameters. First, we attempted to measure the relative amount of mitochondria in different cell lines using qPCR. Surprisingly, the relative mtDNA content was higher in two engineered cell lines which indicates HIPK3 overexpression increased the number of mitochondria (**Figure 7A**). ATP production assay showed that ATP production was in fact higher in wild type cells than HIPK3-overexpressing cells (**Figure 7B**). We further examined protein expression levels of key enzymes of the mitochondrial respiratory chain. Contrary to our expectations, all five respiratory complexes rose upon HIPK3 overexpression, along with another key enzyme in the TCA cycle, citrate synthase (**Figure 7C**). Increase in aerobic respiratory capacity agrees with the finding that ROS production was in fact higher in HIPK3-overexpressing cells. Enhanced mitochondrial oxidative phosphorylation gives rise to elevated ROS production. Quantitative analysis of **Figure 7C** is shown in **Supplementary Figure 3M**.

Aerobic glycolysis is the main source of energy for cancer cells. Although glycolysis it not as efficient as the mitochondrial respiratory chain in terms of ATP production, high intake of glucose, and lactate production which contributes to acidic tumor microenvironment, favor cancer progression. Therefore, measurements of various glycolytic parameters followed. As evident in **Figure 7D**, glucose consumption, lactate production, pyruvate production and Acetyl-CoA all decreased due to HIPK3 overexpression, which converge on a convincing conclusion: HIPK3 overexpression suppresses glycolysis in colorectal cancer. Another type of assay measuring oxygen consumption rates (OCR) and extracellular acidification rates (ECAR) provided direct evidence supportive of a metabolic shift from aerobic glycolysis to aerobic respiration triggered by HIPK3 overexpression. OCRs within the time interval between addition of FCCP and Rotenone + Aptenin indicates maximal respiratory capacity, while ECARs within the time interval between addition of oligomycin and 2-DG indicates maximal glycolytic capacity (**Figure 7E**). As clearly demonstrated in these two graphs, both HIPK3-overexpressing cell lines showed higher maximal respiratory capacity but lower maximal glycolytic capacity than their wild type counterparts (**Figure 7F**).

Once again, we employed qPCR to screen for altered key enzymes engaged in glycolysis, including two isoforms of Hexokinase (HK), two subunits of lactate dehydrogenase (LDH) and two isozymes of pyruvate kinase (PK). Our results indicated that HK2, LDHB and PKM2 transcript levels were lowered in HIPK3-overexpressing cell lines (**Figure 7G**). qPCR results were further confirmed by immunoblotting (**Figure 7H**). Expression of all three glycolytic enzymes was inhibited by HIPK3 overexpression, implicating a regulatory role of HIPK3 in colorectal cancer’s metabolism. Quantitative analysis of **Figure 7H** is shown in **Supplementary Figure 3N**.

### Co-Culturing of Wild-Type Exosomes With HIPK3-Overexpressing Cell Lines Partially Restores Oncogenicity and Glycolytic Ability in Colorectal Cancer Cells

**Figure 8:**
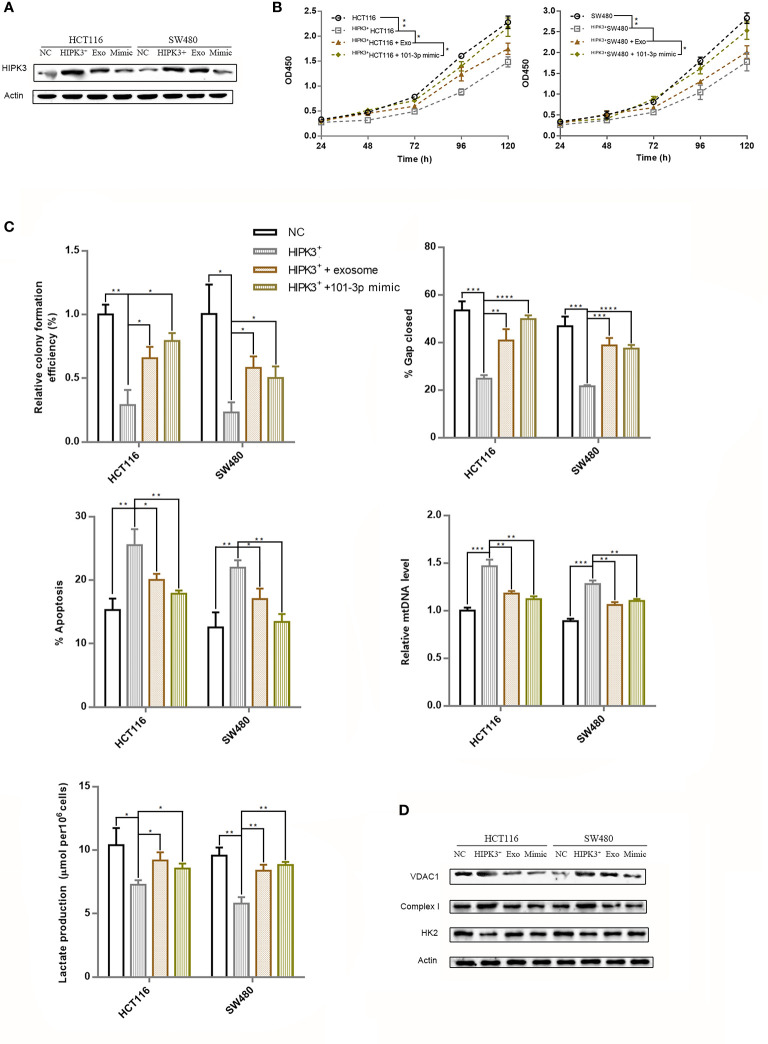
Repression of over-expressed HIPK3 by either wild type exosomes or 101-3p mimic partially restored oncogenicity in HCT116 and SW480 cells. **(A)** Immunoblot of HIPK3 in HCT116 cells stably over-expressing HIPK3 treated either by wild type exosomes or 101-3p mimic. **(B)** Growth curves of HCT116 and SW480 cells stably over-expressing HIPK3 treated either by wild type exosomes or 101-3p mimic. **(C)** Wound healing assay, apoptosis assay, mtDNA, and lactate production assay of HCT116 and SW480 cells stably over-expressing HIPK3 treated either by wild type exosomes or 101-3p mimic. **(D)** Immunoblots of VDAC1, complex I, HK2 in HCT116 and SW480 cells stably over-expressing HIPK3 treated either by wild type exosomes or 101-3p mimic. *, **, ***, **** correspond to a p-value less than 0.05, 0.01, 0.001, and 0.0001, respectively.

We collected exosomes from both wild type HCT116 and SW480 cells and co-cultured them with their respective HIPK3-overexpressing mutant cells. Western blot results showed partial reduction of HIPK3 at the protein level in two HIPK3-overexpressing cell lines co-cultured with exosomes (**Figure 8A**). We also transfected two HIPK3-overexpressing cell lines with 101-3p mimic and observed reduction in HIPK3 protein level as well. Cell growth assay revealed partially restored proliferative potential in two engineered cell lines co-cultured with exosomes as well as those subjected to treatment by 101-3p mimic (**Figure 8B**). Measurements of other parameters, i.e. colony formation, wound healing ability, apoptotic percentage, relative mtDNA level and lactate production, all converge on the finding that wild type exosomes partially recovered oncogenicity and glycolytic capacity of the two HIPK3-overexpressing colorectal cell lines, further reinforcing the finding that exosomes containing 101-3p derived from HCT116 and SW480 can restore oncogenicity and therefore are oncogenic themselves (**Figure 8C**, original data of colony formation and wound healing can be found in **Supplementary Figure 2**). Immunoblots of key proteins in mitochondrial respiration and glycolysis, i.e. VDAC1, complex I and HK2, showed that wild type exosomes and 101-3p mimic altered the expressions of these proteins in HIPK3-overexpressing cells. The way these proteins were altered by wild type exosomes or 101-3p mimic favors oncogenicity (**Figure 8D**).

### Xenograft Model

**Figure 9:**
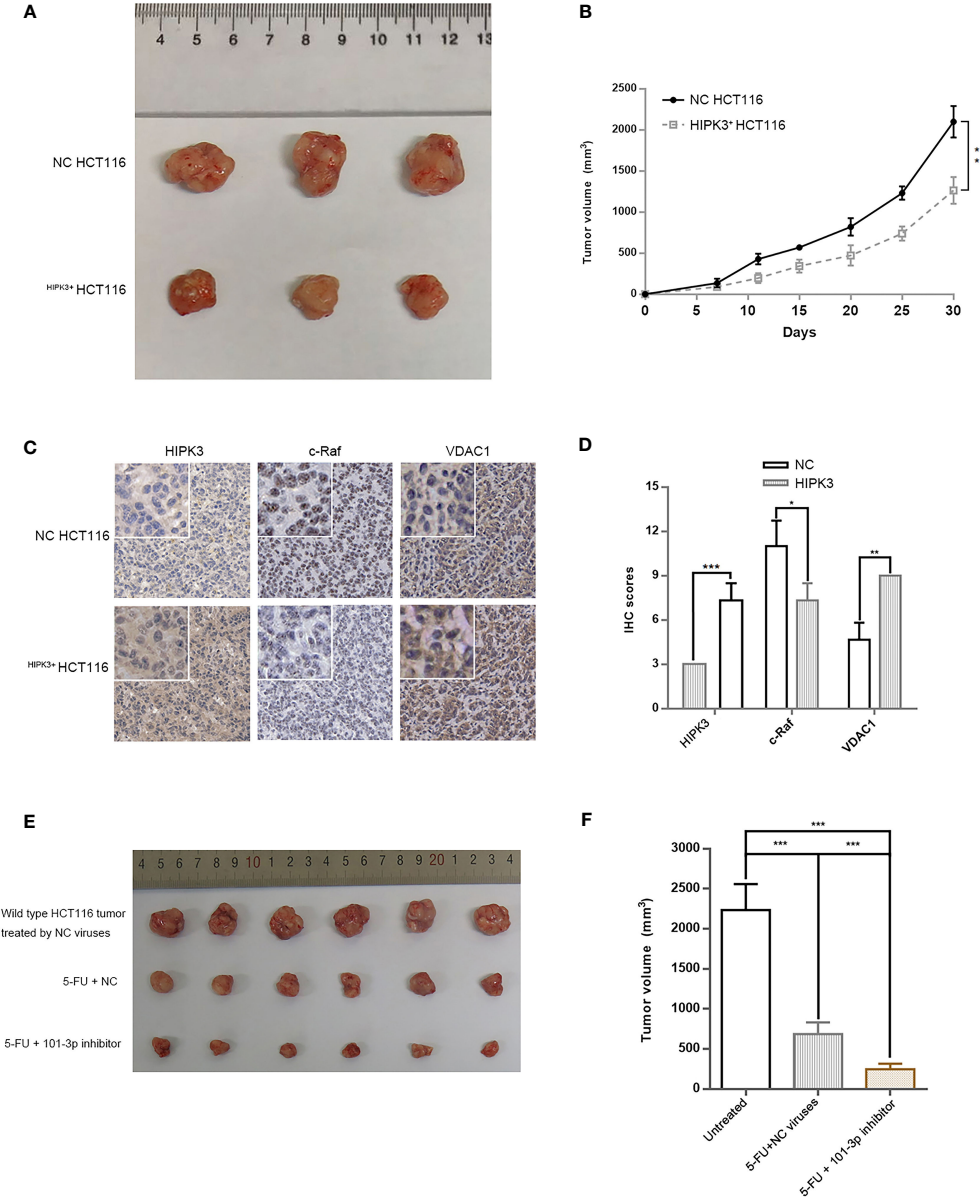
A Combinatorial treatment of 5-FU and 101-3p mimic enhanced anti-tumor efficacy against CRC in nude mouse. **(A)** Tumor formation by HCT116 cells stably over-expressing HIPK3. **(B)** Tumor volume measurements. **(C)** IHC of HIPK3, c-Raf, and VDAC1 in tumors formed by HCT116 cells. **(D)** IHC scores. **(E)** Tumors formed by wild type HCT116 treated by either 5-FU alone, or 5-FU and 101-3p inhibitor. **(F)** Tumor volumes of those shown in **Figure 9E** at day 30. *, **, *** correspond to a p-value less than 0.05, 0.01 and 0.001, respectively.

We transplanted HIPK3-overexpressing and NC HCT116 cells into nude mice for subcutaneous tumor formation. **Figure 9A** shows the size of tumors excised at 30 days post transplantation. Tumors formed by ^HIPK3+^HCT116 cells displayed substantially smaller dimensions compared to NC cells. Tumor growth curve is shown in **Figure 9B**. Tumors were further analyzed by IHC. HIPK3, VDAC1, and c-Raf were stained, and their expressions were observed at the tissue level. HIPK3 expression was strong in HIPK3-overexpressing tumors as expected (**Figure 9C**). c-Raf, which primarily localizes to the nucleus, showed higher expression in NC tumors than HIPK3-overexpressing tumors. Furthermore expression of VDAC1, the mitochondrial pore-forming protein, was elevated in HIPK3-overexpressing tumors. IHC scores of HIPK3, c-Raf, VDAC1 are shown in **Figure 9D**. To exploit the clinical value of 101-3p as a therapeutic target in tumor treatment, we applied a combinatorial treatment of 5-FU and lentiviruses expressing 101-3p inhibitor to xenograft tumors formed by wild type HCT116 (**Figure 9E**). Tumor sizes at 30 days post transplantation were compared among three groups, each with six repeats (**Figure 9E**). The group receiving 5-FU and NC lentiviruses showed smaller tumor size compared to the group treated just by NC viruses. On the other hand, the group receiving 5-FU and lentiviruses expressing 101-3p inhibitor showed even smaller tumor size, with a tumor size reduction of more than 50% compared to the other treatment group. **Figure 9F** shows a direct comparison among these three groups. The synergistic treatment combining 5-FU and 101-3p inhibitor showed significantly improved efficacy compared to 5-FU alone.

## Discussion

In this study, we performed comparative analysis of miRNA expression profiles of clinical samples collected from CRC patients and healthy individuals. hsa-miR-101-3p (101-3p) was conspicuously upregulated in plasma exosome samples in CRC patients compared to healthy individuals. Further studies revealed 101-3p is a oncogenic microRNA whose target is HIPK3, a kinase which phosphorylates the classical apoptotic adaptor protein, FADD. Overexpression of HIPK3 in HCT116 and SW480 suppressed oncogenicity by reducing glycolytic capacity. Therefore, HIPK3 inhibition by 101-3p strengthens oncogenicity in CRC by improving aerobic glycolysis.

Exosomes play critical roles in tumor growth and invasion, e.g. nurturing favorable tumor microenvironment and coordinating a network of cells for cancer progression. A variety of molecules have been identified in exosomes and among which miRNAs are a class of molecules routinely discovered. As gene regulators, delivery of miRNAs to target cell may exert profound effects on gene expressions and therefore modify stereotypic characteristics of target cells. For example, cancer-derived exosomal hsa-miR-25-3p can mediate the formation of pre-metastatic niche by inducing angiogenesis and vascular permeability through silencing KLF2 and KLF4, thereby promoting CRC metastasis (4). Exosomal has-miR-301-3p can induce HIF-1α accumulation and promote GC malignant behaviors and metastasis by targeting PHD3 (25). Many more examples exist to support functional roles of exosomal miRNAs in cancer progression and metastasis.

Cancer has long been treated as a disorder of uncontrolled growth. The six hallmarks of cancer, i.e. sustaining proliferative signaling, evading growth suppressors, resisting cell death, enabling replicative immortality, inducing angiogenesis, and activating invasion, seem to be irrelevant to metabolism (26). However, growing evidence in the last decade has connected the unique metabolic pattern of cancer to all of its invasive features. Although Otto Warburg won the Nobel prize back in 1931, it was not until about 10 years ago did researchers truly begin to understand the importance of glycolysis in cancer. Dependence on the glycolytic bioenergetics is seemingly a sub-optimal strategy for cancer cells, yet it endows cancer with several other advantages, such as acidic microenvironment and avoidance of ROS (27–29). Lactate produced by glycolysis in cancer promotes acidosis and therefore acidification of the tumor microenvironment is now considered to be a direct consequence of the Warburg effect (20, 21). The acidic tumor microenvironment then alters a spectrum of gene expressions in favor cancer progression and malignancy (22, 30, 31).

Our aim was to pinpoint the role of certain exosomal miRNA in colorectal cancer. miRNA sequencing of clinical samples and relevant bioinformatic analysis led us to focus on 101-3p. Inhibiting 101-3p decreased oncogenic potential of HCT116 and SW480. Target analysis of 101-3p led us to further focus on HIPK3, a kinase whose function is to phosphorylate FADD at serine 194, a classical apoptotic adaptor. Besides, circHIPK3 has been implicated in oncogenesis and progression in several types of cancer as described in the Introduction section. However, 101-3p mimic decreased mRNA and protein levels of HIPK3 in both HCT116 and SW480 cells, but not circHIPK3 level. Therefore, according to our study, 101-3p likely to exert its oncogenic potential through inhibition of HIPK3 itself, but not inhibition of circHIPK3. And HIPK3 may exert its tumor suppressive potential through phosphorylation of FADD. Indeed, there are reports which directly linked decreased level of phosphorylated FADD to poor clinical outcomes in cancer (32–36), which support 101-3p’s role as an oncomiR.

Stable overexpression of HIPK3 in HCT116 and SW480 cells by lentivirus was confirmed along with a concomitant elevation in pFADD expression. Stereotypic characterizations of HIPK3-overexpressing cell lines showed HIPK3 is likely a tumor suppressor and sensitizes CRC cell lines to 5-FU.

Based on our results, HIPK3 overexpression is related to increased VDAC1 expression. VDAC1 form mPTPs through which cytochrome C are released upon apoptosis. Increased VDAC1 expression correspond to more mPTPs on the mitochondrial membrane, and therefore enhanced proton leak and increased flux of materials through the mitochondrial membrane. When apoptosis occurs, the ease of cytochrome c release due to increased number of mPTPs is the direct reason for increased sensitivity of HIPK3-overexpressing cells to 5-FU.

Elevation in ROS was also observed in HIPK3-overexpressing cells. Previous studies have reported that opening of mPTPs or increase in VDAC1 expression both lead to increased ROS production. ROS is a subtle companion of cancer as too much or too little ROS both hinder cancer progression. As a byproduct of ATP synthesis in the process of oxidative phosphorylation, ROS generation is necessary for energy production. However cancer cells devise a delicate balance between aerobic respiration and glycolysis to circumvent negative effects exerted by excessive ROS produced by oxidative phosphorylation. HIPK3 overexpression increased ROS generation by increasing VDAC1 expression, and maybe it alters the balance between aerobic respiration and glycolysis as well, which was merely a speculation at this point.

Changes in VDAC1 expression, and several other apoptosis-related factors is just one of many alterations brought by HIPK3 overexpression. The increase in MMP was accompanied by a rise in the number of mitochondria as well as the expression of aerobic respiration-related enzymes. Nevertheless, a faster release of cytochrome c was observed in HIPK3-overexpressing cells. An improved mitochondrial functionality is accompanied by vulnerability to intrinsic apoptosis. This can be explained by increased expression of the pore-forming protein,VDAC1. Heightened expression of VDAC1 leads to formation of more pores formed by VDAC1 oligomerization, and therefore larger efflux of cytochrome c.

HK2 functions in the first and rate-limiting step in glycolysis. Several studies have reported on complexes formed by HK2 and VDAC1 (37–39). HK2/VDAC1 complex provides mitochondrial ATP as a phosphate donor for phosphorylation of glucose to form the glucose-6-phospahte catalyzed by HK2. Although some studies report a concurrent rise and drop in expression of HK2 and VDAC1, our results showed HK2 expression was indeed decreased while that of VDAC1 was increased. Therefore VDAC1/HK2 complex must have been reduced in number, inhibiting the first and vital step in glycolysis. Expression of other key glycolytic enzymes, such as LDHB and PKM2, were also inhibited upon HIPK3 overexpression.

**Figure 10:**
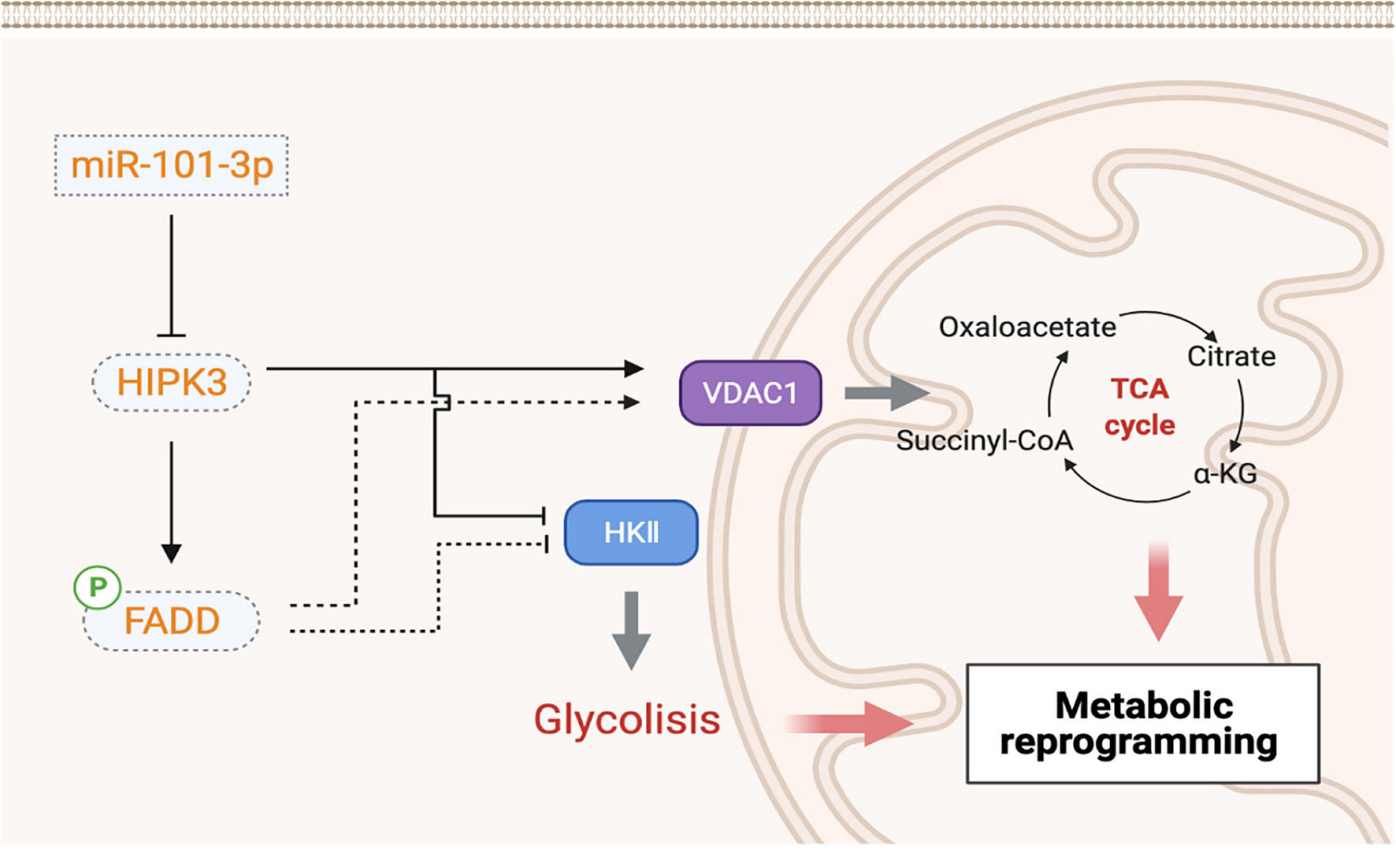
Graphical representation of the 101-3p-HIPK3 axis and its role in CRC oncogenesis.

**Figure 10** shows a demonstration of 101-3p-initiated oncogenic pathway. According to our results, HIPK3, a potential tumor suppressor, is a target of and therefore repressible by 101-3p. HIPK3 promotes FADD phosphorylation and metabolic reprogramming in colorectal cancer. It not yet clear if enhanced FADD phosphorylation is directly linked to metabolic reprogramming and is worthy investigating in the future.

Xenograft experiments showed markedly improved therapeutic potency of 5-FU combined with 101-3p inhibitor. Resistance to chemotherapy has always been a troubling hindrance in cancer treatment. Cocktail treatment has been widely used these days to overcome drug resistance. 101-3p inhibitor weakens colorectal tumors by reprogramming its metabolism, leaving them vulnerable to intrinsic apoptosis induced by chemotherapeutic agents. We believe inhibiting 101-3p or any other means to enhance HIPK3 expression is a feasible approach to sensitize colorectal cancer to chemotherapy.

## Conclusion

This study unraveled an oncogenic nature of the exosomal 101-3p and suggested a relationship between the 101-3p-HIPK3 axis and metabolic homeostasis in colorectal cancer. Expression level of 101-3p is positively correlated with glycolytic capacity in CRC and therefore 101-3p itself is an oncomiR. Combining 101-3p inhibitor with chemotherapeutic agents is an effective strategy against CRC.

## Data Availability Statement

The original contributions presented in the study are included in the article/**Supplementary Material**. Further inquiries can be directed to the corresponding authors.

## Ethics Statement

The studies involving human participants were reviewed and approved by the Ethics Committees of the Nanjing University of Chinese Medicine. The patients/participants provided their written informed consent to participate in this study. The animal study was reviewed and approved by the Ethics Committees of the Nanjing University of Chinese Medicine.

## Author Contributions

LT performed experimental validation assays and drafted the manuscript. CX, WS, and JT performed experiments. LL, MF, and DS analyzed the data. YL designed the study and drafted the manuscript. YL and HC acquired the funding. All authors contributed to the article and approved the submitted version.

## Funding

This study was supported in part by grants from National Natural Science Foundation of China (81930117, 81902330).

## Conflict of Interest

The authors declare that the research was conducted in the absence of any commercial or financial relationships that could be construed as a potential conflict of interest.

## Publisher’s Note

All claims expressed in this article are solely those of the authors and do not necessarily represent those of their affiliated organizations, or those of the publisher, the editors and the reviewers. Any product that may be evaluated in this article, or claim that may be made by its manufacturer, is not guaranteed or endorsed by the publisher.

## Supplementary Material

Supplementary Figure 1
**(A)** Volcano plots of miRNA sequencing results: tumor *vs*. paracancerous tissue. **(B)** Venn diagram of various comparison groups. **(C)** Heat maps of miRNA expressions. The heats maps show all differentially expressed miRNA based on FDR (<0.01) and are arranged in a descending order: from most up-regulated to most-down regulated miRNAs (tumor *vs*. adjacent tissue or CRC plasma exosomes *vs*. Healthy plasma exosomes). 101-3p is indicated by a white arrow. **(D)** Venn diagram of a special comparison: differentially expressed miRNA on the tissue level *vs*. differentially expressed miRNA on the exosomal level.

Supplementary Figure 2
**(A)** Colony formation assay of various cell groups. **(B)** Wound healing assay.

Supplementary Figure 3
**(A–C)** Quantitative analysis of **Figures 1G, I, J**, respectively. **(D–F)** Quantitative analysis of **Figures 3B, D, E**, respectively. **(G, H)** Quantitative analysis of **Figures 4A, B**, respectively. **(I, J)** Quantitative analysis of **Figures 5A, C**, respectively. **(K, L)** Quantitative analysis of **Figures 6C, E**, respectively. **(M, N)** Quantitative analysis of **Figures 7C, H**, respectively. **(O)** Relative expression levels of HIPK3 mRNA and circHIPK3 of HCT116 and SW480 following 101-3p treatment compared to those in untreated counterparts.

## Abbreviations

CRC, colorectal cancer; NC, negative control; qPCR, quantitative real-time polymerase chain reaction; IHC, Immunohistochemistry; MOI, multiplicity of infection; TCGA, The Cancer Genome Atlas.
