# Supplementary figures and images for: HIPK3 Inhibition by Exosomal hsa-miR-101-3p Is Related to Metabolic Reprogramming in Colorectal Cancer
Source: Front Oncol. 2022 Jan 13;11:758336. doi: 10.3389/fonc.2021.758336 (PMC8792385; doi:10.3389/fonc.2021.758336)

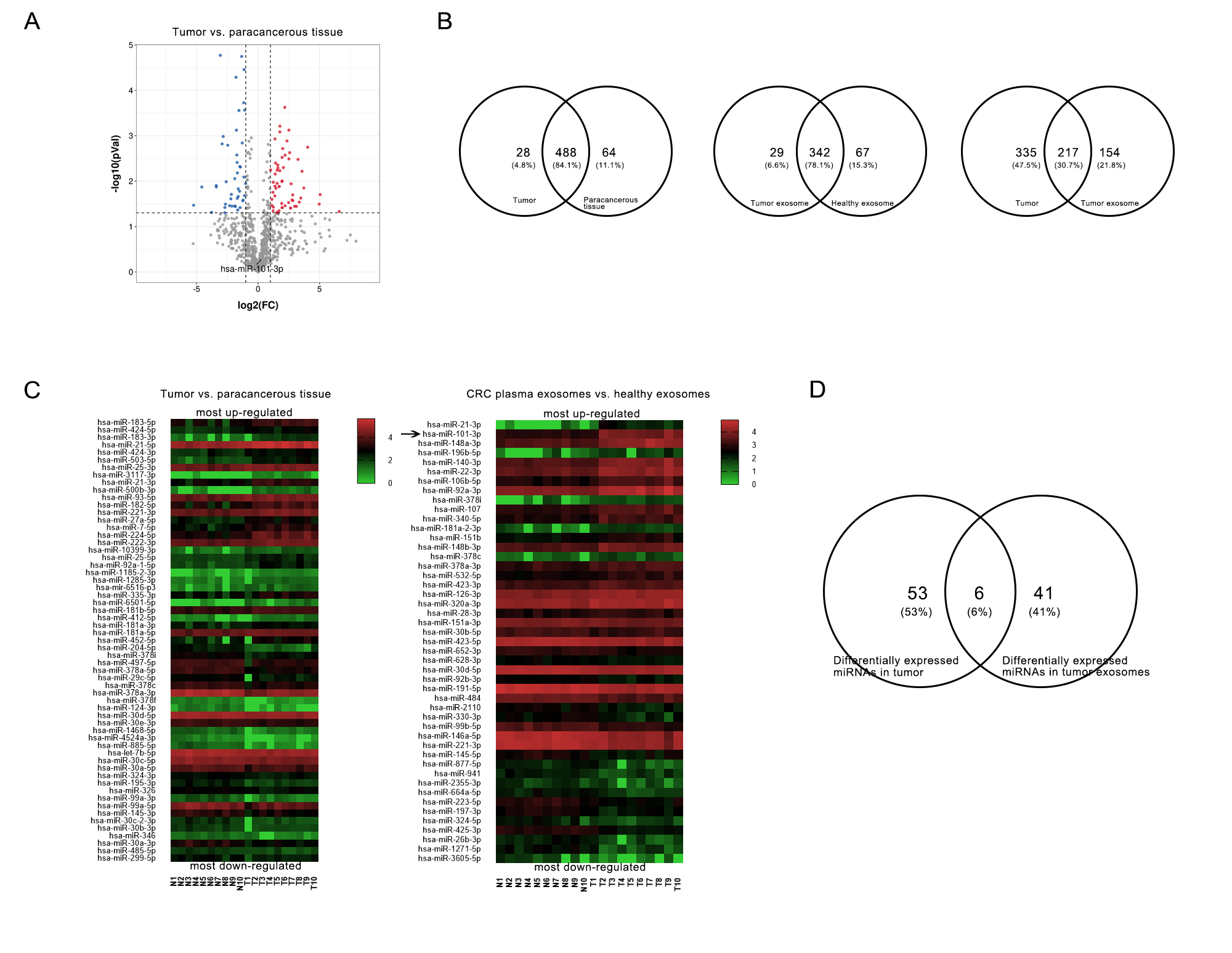

Supplement: Supplementary Figure 1 — (A) Volcano plots of miRNA sequencing results: tumor vs. paracancerous tissue. (B) Venn diagram of various comparison groups. (C) Heat maps of miRNA expressions. The heats maps show all differentially expressed miRNA based on FDR (<0.01) and are arranged in a descending order: from most up-regulated to most-down regulated miRNAs (tumor vs. adjacent tissue or CRC plasma exosomes vs. Healthy plasma exosomes). 101-3p is indicated by a white arrow. (D) Venn diagram of a special comparison: differentially expressed miRNA on the tissue level vs. differentially expressed miRNA on the exosomal level. [file Image_1.jpeg]

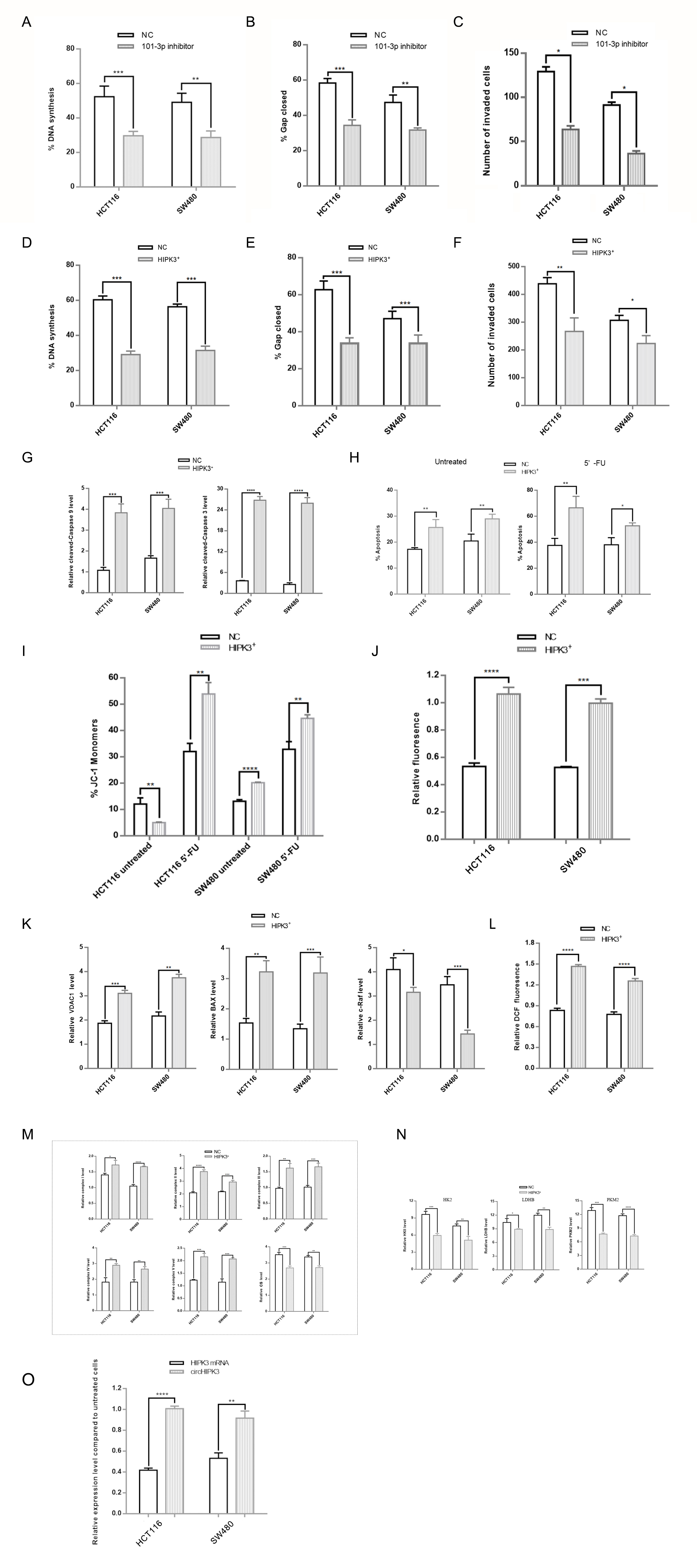

Supplement: Supplementary Figure 3 — (A–C) Quantitative analysis of Figures 1G, I, J , respectively. (D–F) Quantitative analysis of Figures 3B, D, E , respectively. (G, H) Quantitative analysis of Figures 4A, B , respectively. (I, J) Quantitative analysis of Figures 5A, C , respectively. (K, L) Quantitative analysis of Figures 6C, E , respectively. (M, N) Quantitative analysis of Figures 7C, H , respectively. (O) Relative expression levels of HIPK3 mRNA and circHIPK3 of HCT116 and SW480 following 101-3p treatment compared to those in untreated counterparts. [file Image_3.jpg]
