# Supplementary material for: HIPK3 Inhibition by Exosomal hsa-miR-101-3p Is Related to Metabolic Reprogramming in Colorectal Cancer
Source: Front Oncol. 2022 Jan 13;11:758336. doi: 10.3389/fonc.2021.758336 (PMC8792385; doi:10.3389/fonc.2021.758336)
Supplement: Supplementary file 4 [file Table_1.docx]

Supplementary Table S1

Clinical information of CRC patients

| Patient number | Sex | Age | Pathology | TNM stage | |
| --- | --- | --- | --- | --- | --- |
| 1 | Male | 67 | Colon carcinoma | IIIB |  |
| 2 | Male | 69 | Colon carcinoma | IIIB |  |
| 3 | Male | 63 | Colon carcinoma | IIIC |  |
| 4 | Male | 67 | Colon adenocarcinoma | IIIB |  |
| 5 | Male | 60 | Rectal carcinoma | IIIB |  |
| 6 | Male | 55 | Sigmoid colon carcinoma | IVA |  |
| 7 | Male | 60 | Colon carcinoma | IIIC |  |
| 8 | Male | 65 | Sigmoid colon carcinoma | IV |  |
| 9 | Male | 76 | Rectal carcinoma | IIIB |  |
| 10 | Male | 59 | Colon carcinoma | IIIB |  |
