# Supplementary material for: HIPK3 Inhibition by Exosomal hsa-miR-101-3p Is Related to Metabolic Reprogramming in Colorectal Cancer
Source: Front Oncol. 2022 Jan 13;11:758336. doi: 10.3389/fonc.2021.758336 (PMC8792385; doi:10.3389/fonc.2021.758336)
Supplement: Supplementary file 5 [file Table_2.docx]

Supplementary Table S2

| Gene name | Forward primer | Reverse Primer |
| --- | --- | --- |
| Bcl-2 | GGTGAACTGGGGGAGGATTG | GTTCCACAAAGGCATCCCAG |
| Bcl-XL | CCGGGTCGCATGATCCCT | TCTGAAGGGAGAGAAAGAGATTCAA |
| BAX | AAACTGGTGCTCAAGGCCC | AAAGTAGGAGAGGAGGCCGT |
| BAD | TACCTGCCTCTGCCTTCCA | GCCCCAGCGCCTCCAT |
| BAK | GATCCCGGCAGGCTGATCC | AGCTGCGGAAAACCTCCTC |
| BID | AAACAGTCGCGTAAGGGGTCG | CTCTCTGCGGAAGCTGTTGT |
| VDAC1 | GCCCGGAAGGCAGAAGAT | TTGGTGAAGACATCCCTGGC |
| VDAC2 | CTCTTGTGAGAGCGCAAGGT | CTCTCAAGTCCTCGGCCAAT |
| VDAC3 | CACACCAACGTACTGTGACCTA | GACCATGCCAAAGCCATATCCT |
| HK1 | GGACTGGACCGTCTGAATGT | ACAGTTCCTTCACCGTCTGG |
| HK2 | CAAAGTGACAGTGGGTGTGG | GCCAGGTCCTTCACTGTCTC |
| LDHA | ATCTTGACCTACGTGGCTTGGA | CCATACAGGCACACTGGAATCTC |
| LDHB | ATGGCAACTCTAAAGGATCAGC | CCAACCCCAACAACTGAATCT |
| PKM1 | CGAGCCTCAAGTCACTCCAC | GTGAGCAGACCTGCCAGACT |
| PKM2 | ATTATTTGAGGAACTCCGCCGCCT | ATTCCGGGTCACAGCAATGATGG |
| PPARGC1B | TGAGCAGACCTTGACAGTGGAG | GACTATGCTTGATGTCTGGTTTGA |
| RFX3 | AGGAAGTGGTCAACAGACAGGC | GGTAGTACCATCTGGCAGAGAAG |
| GLCCI1 | TAGCTGTTGGGCAGAAGAGGGT | TGTAGTTGCTGCCTCAGTTTGGC |
| DCBLD2 | CCTGCAAAAGCAGTGGACCATG | CTCCTACCAGTGGCTGAGCATA |
| GAB1 | GGAAACTCTTGGCATTCAGGAGG | GCAGTCTGTTTCAGAAGAGGTGG |
| ZBTB34 | GCCAGCTTTCTTCAGATGCAGTG | CTCTTCAGCACCGACGGTAACA |
| PDE4D | GGACACTTTGGAGGACAATCGTG | CCTTTTCCGTGTCTGACTCACC |
| UBE2D1 | GCGCATATCAAGGTGGAGTCTTC | CCATTGTGACCTCAGAATATCGAG |
| EZH2 | GACCTCTGTCTTACTTGTGGAGC | CGTCAGATGGTGCCAGCAATAG |
| TMEM65 | TGCTCAAAGAGCTGCACCGCTT | CCTATGAAAGGTATCGCATTGTGG |
| ZBED4 | AGCGGTTCCATAGCAACGTGCT | GGTGTCATCAAAAGAGGAAGCGC |
| STRN3 | GACTGCTGAAGATGGTGAAGGAG | TGACTTGCCTCCTCCAGATGGA |
| CEP350 | CAAAGCAGCTCAGGTCCATGCA | TCTGTGGTGAGGCGAGCAGTTT |
| UBE2D2 | CTACGATCACAGTGGTCTCCAG | CGAGCAATCTCAGGCACTAAAGG |
| HIPK3 | CGACCTGAGGAGATCAAGCC | ACTTGTGAGGCCATACCTTCC |
| ACTB | CACCATTGGCAATGAGCGGTTC | AGGTCTTTGCGGATGTCCACGT |
| GAPDH | ATGTACGTAGCCATCCAGGC | AGGAAGGAAGGCTGGAAGAG |
| U6 | CTCGCTTCGGCAGCACA | AACGCTTCACGAATTTGCGT |
| hsa-miR-101-3p | GCGCGCGTACAGTACTGTGATA | AGTGCAGGGTCCGAGGTATT |
| circHIPK3 | TATGTTGGTGGATCCTGTTCGGCA | TGGTGGGTAGACCAAGACTTGTGA |
